# H_2_S-Synthesizing Enzymes Are Putative Determinants in Lung Cancer Management toward Personalized Medicine

**DOI:** 10.3390/antiox13010051

**Published:** 2023-12-28

**Authors:** Ana Hipólito, Cindy Mendes, Filipa Martins, Isabel Lemos, Inês Francisco, Fernando Cunha, Teresa Almodôvar, Cristina Albuquerque, Luís G. Gonçalves, Vasco D. B. Bonifácio, João B. Vicente, Jacinta Serpa

**Affiliations:** 1 iNOVA4Health, NOVA Medical School, 1150-069 Lisbon, Portugal; ana.hipolito@nms.unl.pt (A.H.); cindy.mendes@nms.unl.pt (C.M.); filipa.martins@nms.unl.pt (F.M.); a2022507@nms.unl.pt (I.L.); 2 Molecular Pathobiology Research Unit, fromThe Portuguese Institute of Oncology (IPOLFG), 1099-023 Lisbon, Portugal; mfrancisco@ipolisboa.min-saude.pt (I.F.); calbuque@ipolisboa.min-saude.pt (C.A.); 3 Pathology Department, The Portuguese Institute of Oncology (IPOLFG), 1099-023 Lisbon, Portugal; fcunha@ipolisboa.min-saude.pt; 4 Pneumology Department, The Portuguese Institute of Oncology (IPOLFG), 1099-023 Lisbon, Portugal; talmodovar@ipolisboa.min-saude.pt; 5 Institute of Chemical and Biological Technology António Xavier (ITQB NOVA), 2780-157 Oeiras, Portugal; lgafeira@itqb.unl.pt (L.G.G.); jvicente@itqb.unl.pt (J.B.V.); 6 IBB-Institute for Bioengineering and Biosciences, Associate Laboratory i4HB-Institute for Health and Bioeconomy, IST-Lisbon University, 1049-001 Lisbon, Portugal; vasco.bonifacio@tecnico.ulisboa.pt; 7 Bioengineering Department, IST-Lisbon University, 1049-001 Lisbon, Portugal

**Keywords:** cysteine metabolism, cystathionine β-synthase (CBS), cystathionine γ-lyase (CSE), 3-mercaptopyruvate sulfurtransferase (MST), cysteine dioxygenase (CDO1), metabolic plasticity, NSCLC metabolism, metabolism-based therapies

## Abstract

Lung cancer is a lethal disease with no truly efficient therapeutic management despite the progresses, and metabolic profiling can be a way of stratifying patients who may benefit from new therapies. The present study is dedicated to profiling cysteine metabolic pathways in NSCLC cell lines and tumor samples. This was carried out by analyzing hydrogen sulfide (H_2_S) and ATP levels, examining mRNA and protein expression patterns of cysteine catabolic enzymes and transporters, and conducting metabolomics analysis using nuclear magnetic resonance (NMR) spectroscopy. Selenium–chrysin (SeChry) was tested as a therapeutic alternative with the aim of having an effect on cysteine catabolism and showed promising results. NSCLC cell lines presented different cysteine metabolic patterns, with A549 and H292 presenting a higher reliance on cystathionine β-synthase (CBS) and cystathionine γ-lyase (CSE) to maintain H_2_S levels, while the PC-9 cell line presented an adaptive behavior based on the use of mercaptopyruvate sulfurtransferase (MST) and cysteine dioxygenase (CDO1), both contributing to the role of cysteine as a pyruvate source. The analyses of human lung tumor samples corroborated this variability in profiles, meaning that the expression of certain genes may be informative in defining prognosis and new targets. Heterogeneity points out individual profiles, and the identification of new targets among metabolic players is a step forward in cancer management toward personalized medicine.

## 1. Introduction

Lung cancer is a lethal disease responsible for 1.59 million deaths per year worldwide according to the WHO [1]. Although lung tumors are overall molecularly and histologically considered very heterogeneous entities, even within histological subtypes, lung cancer can be classified into two main histological groups: small-cell lung carcinoma (SCLC) and non-small-cell lung carcinoma (NSCLC). NSCLC accounts for around 85% of all lung cancer cases, while SCLC accounts for circa 15% [2,3]. NSCLC is further generally subcategorized as lung adenocarcinoma (LUAD), lung squamous cell carcinoma (LUSC), and large-cell lung carcinoma (LCLC), with the first two being the most commonly diagnosed subtypes [3]. Different factors account for this high mortality, including late diagnosis, molecular heterogeneity, and the development of resistance mechanisms to chemotherapy and targeted therapy [4,5]. Therefore, it is crucial to determine the molecular signatures underlying this chemotherapy response heterogeneity in order to identify appropriate targets and markers to create new treatments and stratify patients. In this line, cancer metabolic remodeling is a hub of information amongst which pivotal players and pathways can be posited as pawns in the design of novel, more specific, and efficient therapies in the scope of personalized medicine.

Metabolic remodeling is employed by cancer cells to adapt and thrive in the organ and tumor microenvironment (TME), and in the last two decades, this field has received attention from the scientific community worldwide [6,7]. Cancer cells rewire their cellular metabolism to sustain the energetic and biosynthetic demands of an elevated proliferation rate, together with the increased capacity of redox control, allowing the metabolic flow [8,9]. Although cancer cells may present different metabolic profiles, they share several characteristics that make specific metabolic pathways important, such as glycolysis; glutamine catabolism; one-carbon metabolism; and redox homeostasis, including cysteine metabolism [8,10,11]. These pathways are dependent on the genetic background of cancer cells, conditioning oncogenic signaling and gene expression. However, the role of TME in the control of nutrients and signaling molecule availability is fundamental [11,12]. Very recently, we demonstrated the relevance of glucose and lactate dynamics in NSCLC, proving that different subsets of NSCLC present distinct metabolic profiles, including glucose versus lactate reliance [13]. Lactate is increasingly accepted as a valuable metabolic source, which is produced due to the high glycolytic rates and managed by cancer cells as a metabolic substrate for oxidative metabolism and gluconeogenesis [10,14,15,16,17]. Lactate has even been considered a major fuel source for the tricarboxylic (TCA) cycle in some NSCLC tumors [18]. Beyond the production of lactate, glycolysis is a supplier of other glucose-dependent pathways, such as the pentose phosphate pathway (PPP), which plays a role in biosynthesis and redox control.

The ability to avoid oxidative-stress-related damage is a powerful skill of cancer cells, which mostly present a metabolic profile, ensuring an efficient free-radical-scavenging capacity involving cysteine-dependent pathways. Our team has disclosed that cysteine bioavailability is pivotal in chemoresistance, and cysteine metabolism is crucial for cancer cell survival [19,20,21]. Cysteine’s impact on cancer cell survival is mainly associated with its role as a component of the antioxidant glutathione (GSH) [22,23,24] and as a substrate for hydrogen sulfide (H_2_S) production [25,26,27,28,29,30,31]. More recently, we documented that cysteine is also a valuable carbon source, being converted into different amino acids, pyruvate, and lactate, pointing out its usefulness as a carbon source for energy and biomass production [21]. Cysteine can be degraded through the activity of cystathionine β-synthase (CBS), cystathionine γ-lyase (CSE), and/or 3-mercaptopyruvate sulfurtransferase (MST), which works together with cysteine aminotransferase (CAT), the latter presenting a mitochondrial and a cytoplasmic form encoded by distinct genes (*GOT1* and *GOT2*, respectively) [32,33,34,35]. CBS, CSE, and MST also catalyze the cyst(e)ine-dependent production of cysteine persulfide (CysSSH), which in several (patho)physiological contexts affords protection from damaging cysteine oxidation [36,37,38]. In addition to these pathways of cysteine enzymatic breakdown to produce H_2_S and organic intermediates, cysteine can be directed to the oxidative metabolism through cysteine dioxygenase (CDO1). Thus, cysteine is converted into cysteine sulfinic acid, leading to the production of taurine, pyruvate and sulfate, which can be channeled to several other metabolic pathways [39,40,41].

All of these enzymes have been somehow implicated in cancer malignancy and poor prognosis, as reviewed by Bonifácio et al. [42]. Among these enzymes, CBS is the most well studied in the cancer context. It acts on cysteine synthesis and degradation, being an important contributor to cancer biology, by degrading cysteine and producing H_2_S, which presents protumoral roles by stimulating bioenergetics and angiogenesis, inhibiting apoptosis, and promoting cell cycle progression [43,44,45]. In fact, CBS has been reported to be aberrantly expressed, accompanied by increased H_2_S levels in lung cancer [26,46,47] and other types of cancer [48,49], being associated with carcinogenesis support and chemoresistance [50]. CBS targeting sensitizes cancer cells to conventional therapy and induces cell death [51].

The aim of this study was to explore cysteine metabolism specificity in different NSCLC in vitro models to better understand disease heterogeneity and to find important candidates to be used as therapeutic targets and prognosis or therapy response markers. Furthermore, we explored the potential of selenium–chrysin (SeChry) as a drug to disturb cysteine availability and metabolism, as we have reported in ovarian cancer [51].

## 2. Methods

### 2.1. Cell Lines and Culture Conditions

Human adenocarcinoma cell line A549 (CCL-185™), mucoepidermoid carcinoma cell line H292 (CRL-1848™), tubular renal cell line HK2 (CRL-2190™), and keratinocyte cell line HaCaT (PCS-200-011™) were obtained from American Type Culture Collection (ATCC, Manassas, VA, USA), and adenocarcinoma cell line PC-9 (90071810) was obtained from European Collection of Authenticated Cell Cultures (ECACC, Porton Down, Salisbury, UK). These NSCLC cell lines present mutations in *EGFR* or *KRAS* as follows: A549 cells (*EGFR* WT (wild-type) and *KRAS* c.34G>A (p.Gly12Ser), H292 cells (*EGFR* WT and *KRAS* WT), and PC-9 cells (*EGFR* exon 19 deletion (Ex19Del; Glu746-Ala750del) and *KRAS* WT). The cell lines represent the main NSCLC histological types LUAD (A549 and PC-9) and LUSC (H292). Cell cultures were performed using Dulbecco’s modified Eagle’s medium 1× (DMEM) (41965-039, Gibco, Life Technologies; Carlsbad, Califórnia, USA) supplemented with 10% fetal bovine serum (FBS; S 0615, Merck), 1% antibiotic–antimycotic (AA; P06-07300, PAN Biotech; Aidenbach, Germany), and 50 μg/mL gentamicin (15750-060, Gibco, Life Technologies). Cells were maintained at 37 °C in a humidified environment with 5% CO_2_, cultured until an optical confluence of 75–100%, and detached with 0.05% trypsin–EDTA 1× (25300-054, Invitrogen; Carlsbad, Califórnia, USA). Cell synchronization was performed under starvation (FBS-free culture medium) overnight prior to in vitro experiment, except for cytotoxicity assays. All experiments were then conducted using 1% FBS medium with the additional supplements stated above. Cells were kept in control conditions or exposed to 0.402 mM L-cysteine (102839, Merck; Darmstadt, Germany).

### 2.2. WST-1 Assay for Cell Metabolic Viability

The WST-1 assay (Roche Applied Science, Indianapolis, IN, USA) was used to evaluate cell metabolic viability. Cells were plated in 96-well clear-bottom tissue culture plates and exposed to different concentrations. Procedures were carried out according to the manufacturer’s instructions. The enzymes of metabolically active cells cleaved the WST-1 reagent into formazan, whose levels were measured by spectrophotometric quantification (450 nm) in a Bio-Rad Laboratories iMark™ microplate absorbance reader (1681130).

### 2.3. Measurement of H_2_S in Cell Homogenates

Cells (5 × 10^5^ cells/well) were seeded in 6-well plates, cultured in control condition, and exposed to experimental conditions of 0.402 mM L-cysteine, 50 μM bromopyruvic acid (BPA; 16490, Sigma-Aldrich; Darmstadt, Germany), and 30 μM sodium hydrosulfide (NaHS;161527, Sigma-Aldrich) for 24 h. Cells were scrapped in PBS 1× and homogenized in NP40 lysis buffer (1% NP40, 150 mM NaCl, and 50 mM Tris-Cl; pH 8.0) on ice for 30 min and centrifuged at 20,000× *g* for 5 min at 4 °C. Cell homogenates (20 μL) were incubated in black 96-well plates with 80 μL of 10 μM 7-azido-4- methylcoumarin (AzMC, L511455, Sigma-Aldrich) with and without 1 mM aminooxyacetic acid (AOAA, C13408, Sigma-Aldrich) and/or 3 mM DL-propargylglycine (PAG, P7888, Sigma-Aldrich), inhibitors of CBS and CSE enzymes. AOAA is an inhibitor of both CBS and CSE, whereas PAG is selective toward CSE.

Protein concentration was determined with the Bradford method (500-0006, Bio-Rad; Berkeley, CA, USA) against a BSA calibration curve. The H_2_S measurements were subsequently normalized to total protein concentration and a blank sample (cellular lysates with and without AOAA, PAG, or both without a probe).

H_2_S levels were monitored following the AzMC probe emission wavelength (λ_exc_: 355 nm; λ_em_: 460 nm) in a VICTOR3 instrument from PerkinElmer (Shelton, Connecticut, USA) and using the Wallac 1420 v3.0 software (Umbelliferone, 0.1 s protocol l). AzMC signal was measured at T = 0, T = 30 min, T = 60 min, T = 90 min, and T = 120 min. T = 0 and average values of these measures were considered for analysis of the results. 

### 2.4. ATP Quantification

Cells were seeded in 6-well plates (5 × 10^5^ cells/well) and cultured in control conditions or exposed to L-cysteine (0.402 mM), and/or 50 μM BPA, and/or 30 μM NaHS for 24 h. Then, cells were scraped in PBS containing 2 mM EDTA, centrifuged at 210× *g* for 5 min, and homogenized in 1% NP40 lysis buffer with 5% protease inhibitor (protease inhibitor cocktail tablets S8830, Sigma-Aldrich) on ice for 30 min. Samples were then centrifuged at 20,000× *g* for 5 min at 4 °C. Protein was quantified with the Bradford method. An ATP determination kit (A22066, Molecular probes) was used in accordance with the manufacturer’s instructions in the presence of 1 mM AOAA and/or 3 mM PAG. The measurements were performed using the luciferase protocol in a VICTOR3 instrument from PerkinElmer/ using the Wallac 1420 v3.0 software (Luminometry, Luciferase FIR protocol). The ATP concentration was determined against an ATP calibration curve within the range of 0 to 30 μM. Assays were performed in biological triplicates.

### 2.5. Nuclear Magnetic Resonance (NMR) Spectroscopy

Nuclear magnetic resonance (NMR) spectroscopy was used to perform the metabolic profiling of NSCLC cell lines. Cells were cultured in 175 cm^2^ T-flasks of H292 (1.5 × 10^7^ cells/flask), A549 (2 × 10^7^ cells/flask), and PC-9 (2.5 × 10^7^ cells/flask) and exposed to control conditions for 24 h. Conditioned culture media was collected and stored at −80 °C.

Cell extracts of methanol/chloroform/water were performed to separate aqueous (methanol/water) and organic (chloroform) phases. The aqueous phase was lyophilized in a SpeedVac Plus system and was suspended in deuterated water (D_2_O) with 0.04% (*v*/*v*) azide, and 0.32 mM 3-(trimethylsilyl)propionic2,2,3,3-*d4* acid (TSP) was used as chemical shift reference and concentration standard. In extracellular metabolites analysis, 30 μL of 3.2 mM TSP and 30 µL of 0.4% (*v*/*v*) sodium azide in D_2_O were added to 540 μL of conditioned culture media (supernatants). ^1^H-NMR spectra were defined at 25 °C in an Ultrashield^TM^ Avance 500 Plus spectrometer (Bruker) with a cryo-prodigy TCI-Z probe using a noesypr1d pulse program. Spectra acquisition and processing were performed using the TopSpin 4.1 software (Bruker). The assignments were performed by resorting to spectral databases. Metabolite concentrations were determined using Human Metabolome (HMDB) and Chenomx NMR Suite 8.11. 

### 2.6. Quantitative Real-Time PCR (qPCR)

From the cell lines, total RNA was extracted using RNeasy Mini extraction kit (74104, Qiagen, Hilden, Germany) and cDNA synthesized from 1 μg RNA by SuperScript II reverse transcriptase (18080044, Thermo Fisher Scientific; Carlsbad, CA, USA), both according to the manufacturer’s protocol. 

From tumor samples, RNA was extracted from FFPE (formalin-fixed paraffin-embedded) sections of NSCLC samples collected in Instituto Português de Oncologia de Lisboa Francisco Gentil, EPE (IPOLFG, EPE) upon semiautomatic isolation with the Maxwell purification system (Maxwell RSC RNA FFPE Kit, AS1440, Promega). Complementary DNA (cDNA) was synthesized as mentioned above. SYBR Green PCR master mix (04707516001, Roche) was used according to the manufacturer’s protocol in quantitative real-time PCR (qPCR). The genes quantified and the specific primers are presented in Table 1. 

Real-time PCR was carried out during 40 amplification cycles according to the manufacturer’s instructions using a Lightcycler^®^ 480 System instrument (05015243001, Roche). Hypoxanthine-guanine phosphoribosyltransferase (HPRT) was used as a housekeeping gene (Table 1). Experiments were performed in biological triplicates. 

### 2.7. Immunofluorescence

Cells (1 × 10^5^ cells/well) were seeded in glass cover slips in 24-well plates coated with 0.2% gelatin from porcine skin (G-1890, Sigma-Aldrich). Cell fixation was performed with 2% paraformaldehyde for 15 min at 4 °C, after which the cells were incubated with 50 mM ammonium chloride (NH_4_Cl) for 10 min. After blocking for 1 h with PBS 1×, 0.5% BSA, 0.1% saponin-PBS (*w*/*v*/*v*), cover slips were incubated with primary antibodies (Table 2) diluted in PBS 1×, 0.5% BSA, 0.1% saponin-PBS (*w*/*v*/*v*) overnight at 4 °C. Cells were incubated with secondary antibody for 2 h at room temperature (1:1000; Alexa Fluor^®^ 488 goat antirabbit, A-11034, Thermo Fisher Scientific; 1:1000; Alexa Fluor^®^ 488 goat antimouse, A-11001, Thermo Fisher Scientific; Alexa Fluor^®^ 594 goat antimouse, A-11032, Thermo Fisher Scientific; 1:1000). Between incubations, cells were rinsed twice with PBS 1× for 5 min.

Slides were mounted in VECTASHIELD media with DAPI (4′-6-diamidino-2-phenylindole) (H-1200-10, Vector Labs) and evaluated by standard fluorescence microscopy under a Zeiss Imager.Z1 AX10 microscope. The *CytoVision 7.0* software was used for image acquisition and processing, and the *ImageJ 1.54* software was used for signal quantification. 

### 2.8. Proliferation Curves

Cell proliferation rate was determined using proliferation curves. Cells were cultured in 24-well plates (5 × 10^4^ cells/well) in complete DMEM, and after synchronization upon starvation, cells were exposed to the experimental conditions. At each time point (0, 6, 10, 24, 32, and 48 h), cells were detached as described (supernatant was also collected) and centrifuged for 5 min at 155× *g*. The supernatant was discarded, and the cells were counted by staining with Trypan Blue stain 0.4% (15250061, Thermo Fisher Scientific) to identify cells with a compromised cell membrane, hence indicating cell death, using a Neubauer improved cell-counting chamber.

### 2.9. Bioinformatic Analysis

LUAD and LUSC RNA-Seq data from the Cancer Genome Atlas (TCGA) [52] were analyzed using the cBio Cancer Genomics Portal (http://cbioportal.org, accessed on 13 May 2023) [53,54] to explore a LUAD and LUSC genomic dataset that included *CBS*, *CTH*, *MPST*, *GOT1*, *GOT2*, *CDO1*, *SLC1A1*, and *SLC7A11*.

### 2.10. Next-Generation Sequencing for Mutation Detection 

Somatic mutational data for *EGFR*, *KRAS*, *BRAF*, *ALK*, *ROS1*, and *MET* genes was obtained from 55 NSCLC samples in the context of the mutation analysis performed for clinical management according to standard guidelines (IPOLFG Ethical Committee Ref: UIC/1442). The analysis was performed by next-generation sequencing (NGS) using the Ampliseq^TM^ Focus Panel kit (Illumina) on the Miseq platform (Illumina) considering a minimum depth coverage of 500×. The bioinformatics analysis was conducted with the *DNA + RNA Amplicon v.1.0.5* software (Illumina) for alignment and the *Alissa Interpret v.5.3.4* (Agilent Technologies) for analysis and annotation of the genetic variants.

### 2.11. Statistical Analysis

Statistical analysis and EC_50_ calculation were performed using the *GraphPad Prism 8.0* software (www.graphpad.com). Sample data were presented as mean (normal distribution) ± SD. Assays were performed with biological triplicates per treatment. A two-tailed unpaired Student’s *t*-test was used for comparisons between groups, and one-way or two-way ANOVA was used for multiple comparisons. In samples from NSCLC tumor, the assessment of differences in gene expression was performed with 2-sided independent samples (Kruskal–Wallis one-way ANOVA with multiple comparisons or Mann–Whitney test) considering the adjusted significance. The existence of a linear relationship between two variables was assessed using a two-tailed Spearman correlation test. Differences between experimental conditions were considered statistically significant at *p* < 0.05. Multivariate statistical analysis of ^1^H-NMR data was performed on *MetaboAnalyst 5.0* (assessed on 16 November 2022) using metabolites concentrations as inputs and scaled using pareto-scaling. Heatmaps related to the univariate analysis of intracellular and extracellular metabolite levels, detected by NMR, were created with the *GraphPad Prism 8.0* software.

## 3. Results

### 3.1. NSCLC Cell Lines Rely on Cysteine for H_2_S and ATP Production

**Figure 1:**
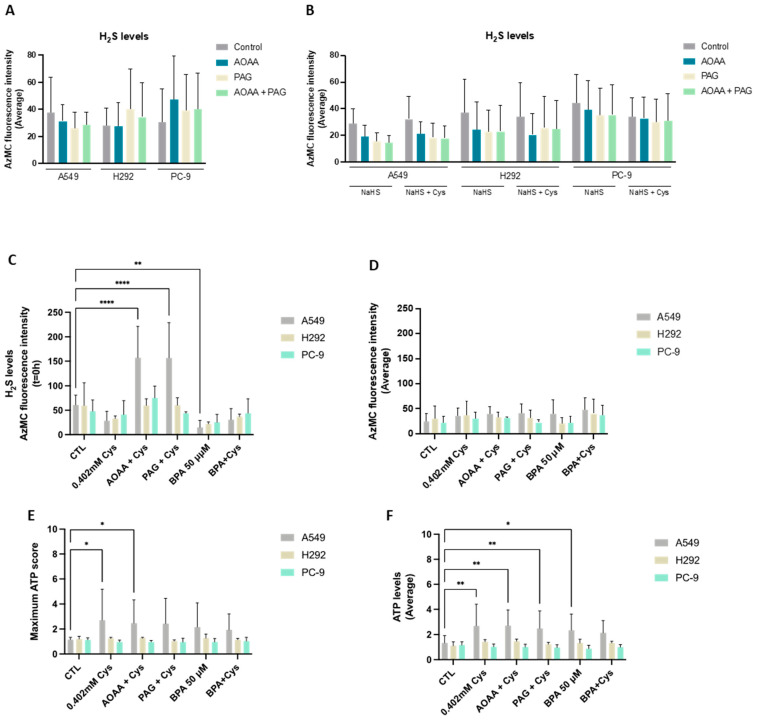
NSCLC cell lines present cysteine reliance on H_2_S production. (**A**) NSCLC cell lines showed similar basal levels of H_2_S production. To verify if H_2_S production was directly dependent on cysteine degradation, cells were exposed to AOAA/PAG treatment. (**B**) NSCLC cells cultured in the presence of the H_2_S donor NaHS alone and in combination with cysteine showed that H_2_S levels decreased when exposed to AOAA and PAG. Upon cysteine treatment, PC-9 showed a tendency to decrease the average levels of H_2_S in control conditions and compensated production of H_2_S upon exposure to inhibitors. (**C**) Upon cysteine supplementation, NSCLC cell lines tended to decrease H_2_S production at T = 0 h but compensated the CBS and CSE inhibition, maintaining (H292 and PC-9) or increasing (A549) H_2_S levels upon AOAA and PAG exposure. Analysis of H_2_S at T = 0 h indicated that A549 and H292 cell lines presented decreased levels of H_2_S in the presence of cysteine with and without glycolysis inhibition with BPA, while the PC-9 cell line presented no significant changes in all conditions. (**D**) Analysis of H_2_S levels showed that all cell lines presented a similar basal ability to produce H_2_S. Maximum score (**E**) or the average levels (**F**) of ATP production indicated that A549 cells increased the production of ATP upon cysteine supplementation independently of the presence of inhibitors, while H292 and PC-9 were able to maintain ATP levels in all experimental conditions. Maximum ATP score and average levels indicated that A549 cells increased the levels of ATP upon BPA and/or cysteine supplementation, and H292 and PC-9 cells maintained the ATP levels in all culture conditions independently of glycolysis inhibition. All data were normalized to the control condition and are represented as mean ± SD. * *p* < 0.5, ** *p* < 0.01, **** *p* < 0.0001.

Cysteine catabolism is an important pathway in biomass and energy production, and metabolic routes accounting for the generation of H_2_S and organic compounds are the most explored in cancer [42]. Thus, we started by evaluating the efficacy of NSCLC cell lines in reductive cysteine catabolism through the measurement of H_2_S levels. Interestingly, all cell lines presented similar basal H_2_S levels (Figure 1A). In an attempt to correlate H_2_S levels with reductive cysteine degradation, the inhibition of CBS and CSE enzymes by AOAA and PAG was analyzed, revealing no significant differences. An absent effect was also observed when NSCLC cells were cultured in the presence of an H_2_S donor (NaHS) alone and in combination with cysteine (Figure 1B).

As NaHS may have prevented fully appreciating the effect of cysteine, H_2_S levels were also measured in cells exposed to cysteine supplementation in the absence of NaHS (Figure 1C,D). In A549 and H292, cysteine supplementation tended to decrease H_2_S levels at 0 h (T = 0 h), whereas PC-9 remained practically unaltered (Figure 1C). Notably, cysteine supplementation in the presence of AOAA increased H_2_S levels at T = 0 h in A549 compared to cysteine alone when exposed to AOAA, PAG, or BPA. The same was observed for cysteine supplementation in the presence of PAG for the A549 and H292 lines. Despite these differences in H_2_S levels at T = 0 h, the steady-state average levels remained unaltered between culture conditions (Figure 1D). Importantly, as H_2_S can be an electron donor to the electron transport chain (ETC) via sulfide:quinone oxidoreductase, accounting for ATP production [55], ATP levels were measured. Considering both the ATP production at T = 0 h and the steady-state average levels, A549 cells increased the production of ATP upon cysteine supplementation independently of the presence of inhibitors (Figure 1E,F). H292 and PC-9 were able to maintain ATP levels in all experimental conditions (Figure 1E,F). Thus, all cell lines presented a high metabolic adaptive capacity.

In order to understand if cysteine metabolism is a core energetic circuitry in NSCLC and because we recently documented that glucose-dependent pathways were pivotal in NSCLC [13], experiments blocking glycolysis with BPA with and without cysteine supplementation were performed by measuring H_2_S and ATP levels. The A549 cell line presented decreased H_2_S levels in the presence of cysteine with and without glycolysis inhibition with BPA (Figure 1C). This decrease was even more evident when comparing BPA alone with control conditions. The PC-9 cell line presented no statistically significant changes in all conditions (Figure 1C,D). Interestingly, A549 cells tended to increase ATP levels upon BPA and/or cysteine supplementation (Figure 1E,F), and H292 and PC-9 cells maintained ATP levels in all culture conditions independently of glycolysis inhibition (Figure 1E,F). Therefore, these results suggest that glucose-dependent energetic pathways, which are abrogated by BPA, can be compensated in part by cysteine-dependent H_2_S production or that glycolysis is preferably employed for synthetic pathways instead of energetic ones.

### 3.2. The Differential Expression of Cysteine Metabolic Players Corroborates Heterogeneous Metabolic Profiles

**Figure 2:**
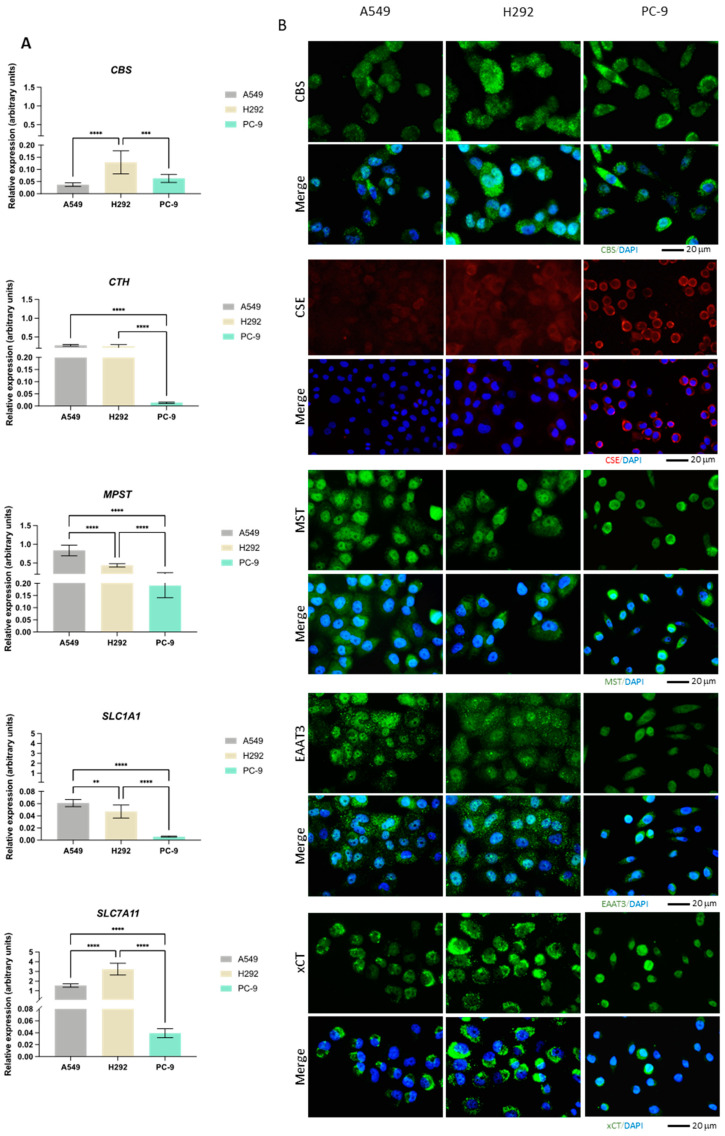

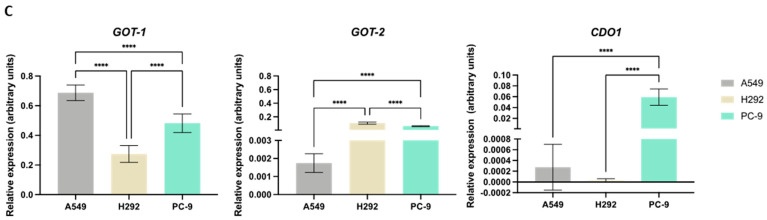
NSCLC cells express distinct expression patterns of enzymes and transporters involved in cysteine metabolism. (**A**) mRNA expression level analysis showed that H292 cells expressed noteworthy levels of *CBS* and *SLC7A11* compared to A549 cells, while PC-9 cells showed low overall expression of *CTH*, *MPST*, *SLC1A1,* and *SLC7A11*. (**B**) Immunofluorescence analysis showed that protein expression followed mRNA expression patterns, reporting similar differences. (**C**) mRNA expression level analysis further showed that H292 cells highly expressed *GOT2*, while PC-9 showed high expression levels of *GOT2* and *CDO1* compared to A549 cells. All mRNA expression level data are relative to *HPRT1* and represented as mean ± SD. ** *p* < 0.01, *** *p* < 0.001, **** *p* < 0.0001.

The expression of *CBS*, *CTH*, *MPST*, *GOT1*, *GOT2*, *CDO1*, *SLC1A1*, and *SLC7A11* genes was evaluated by quantification of the relative mRNA (Figure 2A). Additionally, the expression of CBS, CSE, and MST enzymes as well as EAAT3 and xCT transporters was measured by immunofluorescence (Figure 2B). While *CBS* transcriptional levels were higher in H292, the protein levels detected by immunofluorescence were similar among the cell lines. Moreover, CBS appeared to be both diffusely localized and in small puncta. *CTH* mRNA levels were significantly lower in PC-9 than in the other cell lines, which was not translated into the protein levels. While CSE appeared diffusely expressed in A549 and H292 cells, in PC-9, the protein appeared to localize closer to the membrane. *MPST* mRNA levels were highest in A549 with respect to the other cell lines, with PC-9 exhibiting the lowest mRNA levels. MST expression was similar to CBS (diffuse and in puncta), with comparable protein levels among the three cell lines. *SLC1A1* and *SLC7A11* mRNA levels were relatively low in all cell lines and, similarly to *CTH*, much lower in PC-9 compared to A549 and H292. However, this did not translate to EAAT3 and xCT protein levels, which were similar in all cell lines and equally exhibited enriched signal in puncta. 

While *GOT1* had higher mRNA levels in A549 compared to PC-9 and particularly H292, *GOT2* showed the lowest levels in A549 (Figure 2C). *CDO1* had overall relatively low mRNA levels in all lines, with PC-9 exhibiting the highest levels and *CDO1* mRNA being practically undetectable in H292.

Overall, these expression and localization patterns indicate that heterogeneous metabolic profiles may be found among these NSCLC cell lines, with cysteine metabolism relying on different routes, likely to serve different metabolic requirements.

**Figure 3:**
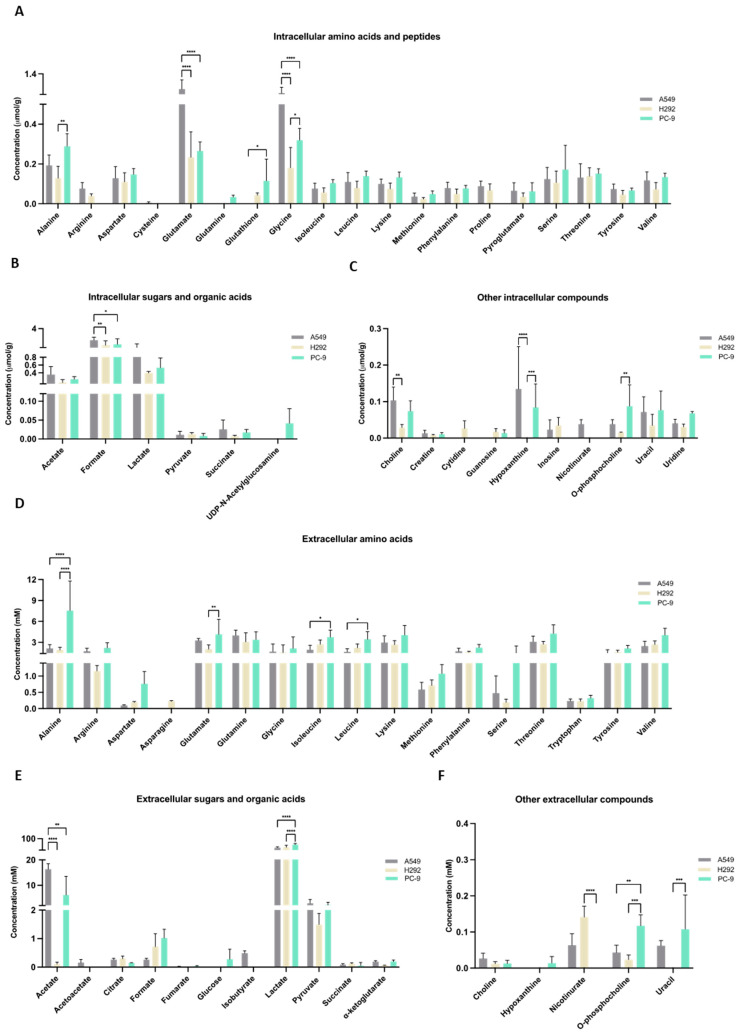

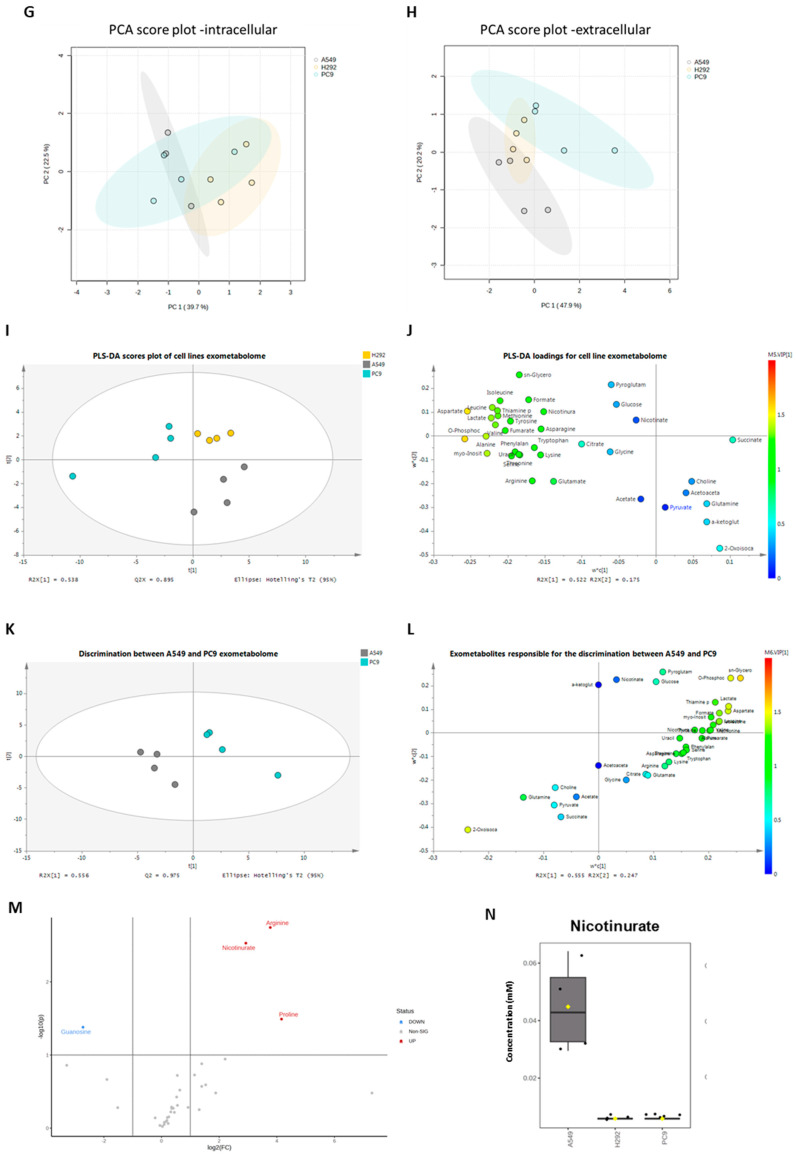
Distinct molecular backgrounds induce individual metabolic patterns in NSCLC cells. (**A**–**C**) ^1^H-Nuclear magnetic resonance (NMR) of the NSCLC panel studied indicated alterations between A549, H292, and PC-9 regarding levels of intracellular amino acids and peptides, sugars and organic acids, and other metabolites. (**D**–**F**) NMR of the NSCLC panel studied further indicated alterations between A549, H292, and PC-9 regarding levels of extracellular amino acids and peptides and sugars and organic acids. Data are represented as mean ± SD. * *p* < 0.5, ** *p* < 0.01, *** *p* < 0.001, **** *p* < 0.0001. (**G**,**H**) PCA showed no differences in the endometabolome (**G**), but the exometabolome (**H**) indicated that A549 and PC-9 cells present distinct metabolic profiles, and H292 cells had common metabolic patterns with A549 and PC-9 cells. (**I**) PLS-DA analysis of the exometabolome allowed the discrimination of the three cell lines. From the PLS-DA analysis of the metabolites present in the cell media, it was possible to discriminate PC-9 from the other cell lines in the first component, while A549 could be discriminated in the second component (upper panel), Q^2^ = 0.873. (**J**) The loading plot (lower panel) showing the metabolites important for the discrimination, colored by VIP in the first component. 2-Oxoisocaproate increased in A549, while aspartate, lactate, phosphocholine, and glycerophosphocholine increased in PC-9, making them important for discrimination between these cell lines. (**K**) From the PLS-DA analysis of the metabolites present in the cell media, it was possible to discriminate between A549 and PC-9 cell lines (upper panel), Q^2^ = 0.975. (**L**) The loading plot (lower panel) showing the metabolites important for the discrimination, colored by VIP in the first component. (**M**) Metabolites significantly different between PC-9 and A549. Volcano plot (fold change >2 and *p* value < 0.05) of the intracellular metabolites between A549 and PC-9. Arginine, proline, and nicotinurate were significantly increased in A549 and guanosine in PC-9. (**N**) Nicotinurate was only present in A549 cells. Box plot of nicotinurate intracellular concentrations in the three cell lines (ANOVA analysis *p* value = 0.00028097 × 10^4^.

The metabolic profiles were defined by NMR spectroscopy, and focus was given to metabolites related to cysteine metabolism. Within cells, very little amount of cysteine was detected and only in A549 cell extracts (Figure 3A). Cysteine was not detected in the culture supernatants of any cell line (Figure 3D), suggesting that the cells consumed cysteine. Cystine was not detected in the cell extracts or supernatants. GSH was not detected in A549 cells, but H292 and PC-9 presented similar amounts of GSH (Figure 3A). Regarding intracellular glutamate, glutamine, and serine levels, A549 showed higher glutamate levels than H292 and PC-9, glutamine was not detected in A549 and H292 cell lines and were only present in trace levels in PC-9, and no differences were observed in intracellular serine levels (Figure 3A). In the supernatants, PC-9 presented the highest levels of glutamate and serine, while glutamine was found in similar amounts in all cell lines (Figure 3D). No differences between cell lines were found in intracellular lactate and pyruvate levels (Figure 3B). In the supernatants, PC-9 cells presented the highest levels of lactate, and no differences were seen in pyruvate levels (Figure 3E). Additionally, extracellular acetate levels were significantly lower in H292 compared to A549 and PC-9 (Figure 3E). Intracellular levels of choline, hypoxanthine, and *o*-phosphocholine presented disparities between cell lines (Figure 3C). Extracellular levels of nicotinurate, *o*-phosphocholine, and uracil were also quite different between cell lines (Figure 3F). Interestingly, in the intracellular extracts, nicotinurate was only detected in A549 cells, making it a distinctive metabolite (Figure 3C,N). A principal component analysis (PCA) that considered all the detected intracellular metabolites showed no differences in the endometabolome (Figure 3G), but the exometabolome (extracellular metabolites) indicated that A549 and PC-9 cells presented distinct metabolic profiles and H292 cells shared common metabolic patterns with A549 and PC-9 cells (Figure 3H). 

The PLS-DA analysis showed the relevance of metabolites in the exometabolome, highlighting the differences between all cell lines (Figure 3I,J). The discrimination between PC-9 and the other two cell lines relied on higher levels of aspartate and *o*-phosphocholine, while the discrimination between A549 and H292 relied on higher levels of pyroglutamate and *sn*-glycero-phosphocholine in H292 and 2-oxoisocaproate and α-ketoglutarate in A549. Considering only A549 and PC-9, as these cell lines presented a different exometabolome in PCA analysis, the PLS-DA analysis strengthened the differences and the role of the previous metabolites in the discrimination between these two cell lines (Figure 3K,L). A volcano plot of the endometabolites indicated that guanosine, nicotinurate, arginine, and proline were the major endometabolites contributing to the distinction between A549 and PC-9 cell lines (Figure 3M). Importantly, nicotinurate was significantly increased in A549 compared to the other cell lines (Figure 3N).

### 3.3. NSCLC Cell Lines Are Chemoresistant, and SeChry Reduces the Metabolic Viability of A549 and H292 Cells, Which Seem to Present a Higher Metabolic Reliance on Cysteine Degradation by CBS

**Figure 4:**
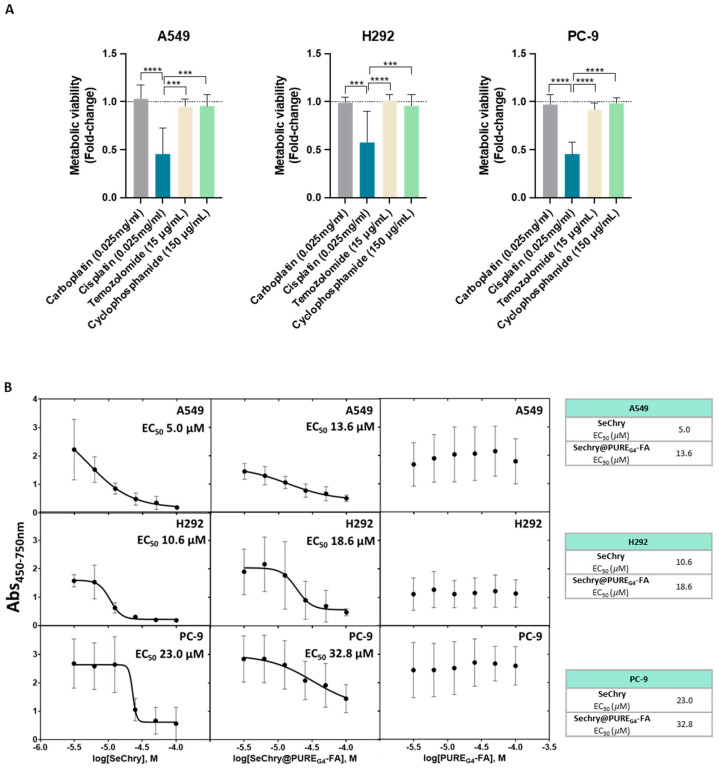

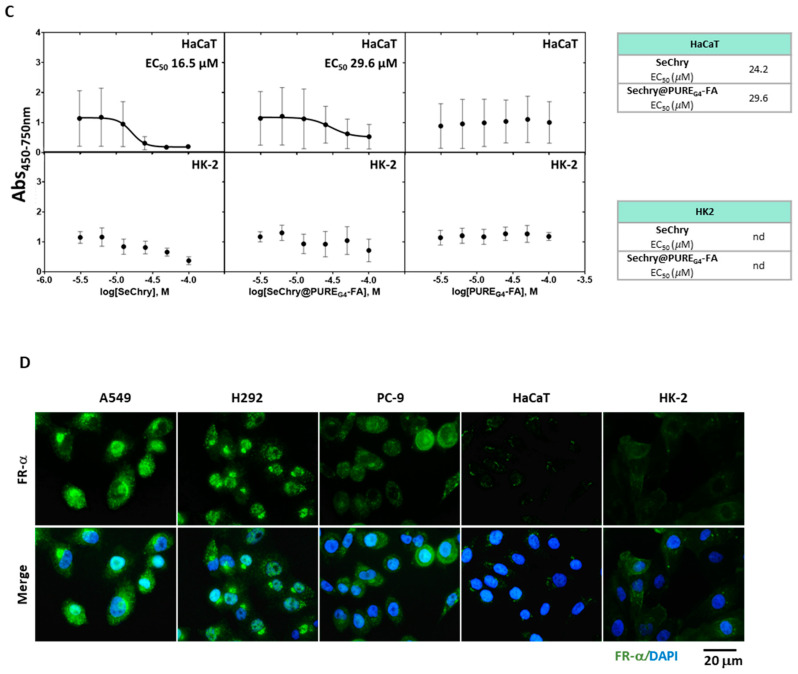
NSCLC cell lines are chemoresistant, and SeChry@PURE_G4_-FA induces decreased cell viability in NSCLC cells, with specificity toward tumor cells rather than nontumoral cells. (**A**) NSCLC cell lines exposed to the most commonly used therapy regimens showed overall resistance to these drugs, except for cisplatin. (**B**) EC_50_ curves and values for SeChry, Sechry@PURE_G4-_FA, and PURE_G4-_FA in NSCLC indicated a higher sensitivity of A549 and H292 to the treatment than PC-9. Empty nanoparticles (PURE_G4._FA) did not induce relevant toxicity on cell viability. (**C**) EC_50_ curves and values for SeChry, Sechry@PURE_G4-_FA, and PURE_G4-_FA in noncancer cell lines (HaCaT and HK2) indicated no effect on cell viability. Empty nanoparticles (PURE_G4._FA) did not induce relevant toxicity on cell viability. (**D**) Detection of FR-α by immunofluorescence indicated high protein levels in NSCLC but not in nontumoral cell lines. FR- α is labeled in green, and nuclei were counterstained with DAPI (blue). Magnification 400×, scale 20 µm. *** *p* < 0.001, **** *p* < 0.0001.

Knowing that cysteine metabolism impairs conventional drugs’ anticancer efficacy [20], metabolic viability was evaluated in NSCLC cell lines exposed to the most commonly used drugs in clinical regimens for NSCLC. Interestingly, cisplatin was the most deleterious drug in all cancer cell lines, having a higher impact on cell viability loss (Figure 4A). Because all cell lines presented resistance to at least three drugs used in conventional NSCLC therapy, they were considered chemoresistant (Figure 4A). 

Our previous studies on ovarian cancer showed that selenium–chrysin (SeChry) anticancer effects were related to cysteine metabolic disruption due to CBS inhibition and GSH depletion [51]. Thus, metabolic viability assays were performed to evaluate the impact of SeChry on cysteine metabolic reliance in NSCLC cell lines. SeChry was tested in a free form and encapsulated in a folate conjugate dendrimer nanoparticle to target cancer cells (SeChry@PURE_G4_-FA), as described previously [54,56]. 

In order to disclose the putative cytotoxic effects on noncancer cells, tubular renal cells (HK2) and keratinocytes (HaCaT) were also tested in parallel with NSCLC cell lines. All NSCLC cell lines, except PC-9, were quite sensitive to SeChry and SeChry@PURE_G4_-FA (Figure 4B). Amongst the NSCLC cell lines, PC-9 presented the highest half-maximum effective concentration (EC_50_) for SeChry and SeChry@PURE_G4_-FA (Figure 4B), suggesting PC-9 may not have a metabolic reliance on cysteine involving CBS, which fits with H_2_S and ATP levels described above. EC_50_ values regarding the SeChry effect on noncancer cells were quite high, whereas EC_50_ for SeChry@PURE_G4_-FA was impossible to determine (Figure 4C). Regarding the expression of folate receptor alpha (FR-α), A549 and H292 expressed high levels and PC-9 cells showed low levels of FR-α, and it was almost undetectable in nonmalignant cells (Figure 4D). The empty nanoparticles did not have any effect on the metabolic viability of any NSCLC (Figure 4B) and noncancer (Figure 4C) cell lines. Free and encapsulated SeChry also decreased all NSCLC cell lines and HK2 proliferation, while no effect was observed in HaCaT (Supplementary Figure S1).

### 3.4. Expression Profile of Genes Involved in Cysteine Metabolism Can Help Stratifying Patients

**Figure 5:**
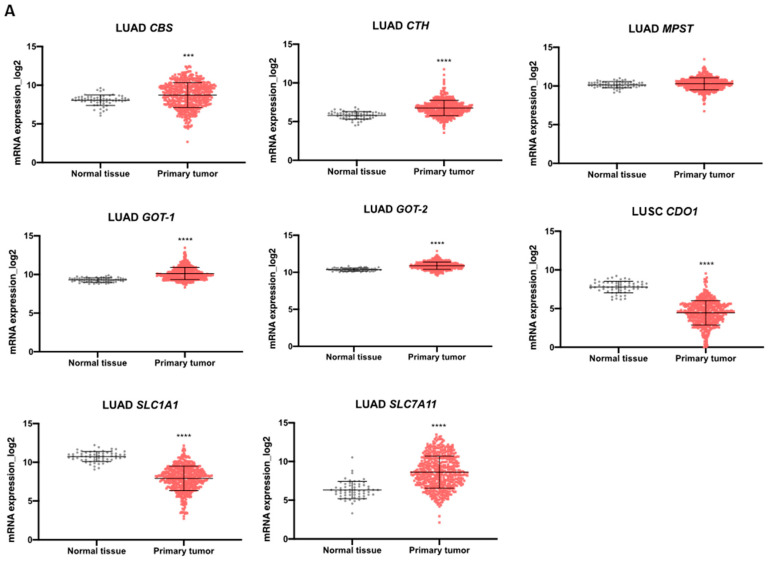

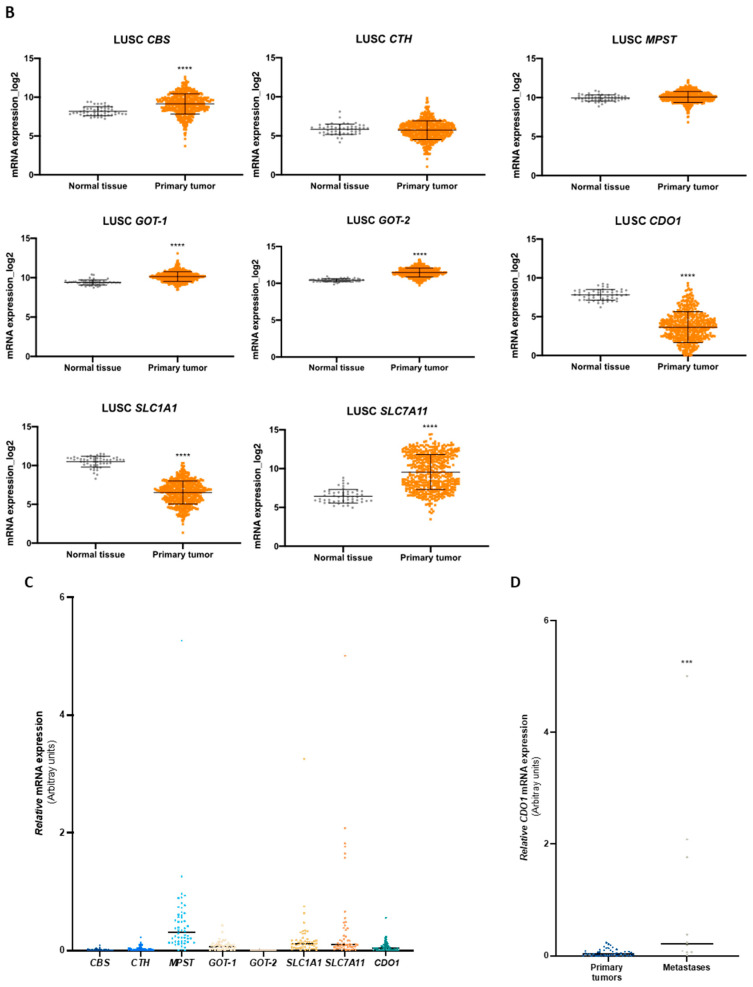
Enzymes and transporters of cysteine metabolism are overexpressed in NSCLC patients. (**A**) Analysis of mRNA levels showed upregulation of *CBS*, *CTH*, *GOT1*, *GOT2,* and *SLC7A11* and a significant downregulation of *SLC1A1* and *CDO1* in NSCLC primary tumors of the LUAD TCGA cohort. (**B**) Analysis of mRNA levels showed upregulation of *CBS*, *GOT1*, *GOT2*, and *SLC7A11* and a significant downregulation of *SLC1A1* and *CDO1* in NSCLC primary tumors of the LUSC TCGA cohort. (**C**) Analysis of the expression of genes encoding H_2_S-producing enzymes in the IPOLFG NSCLC cohort indicated that *MPST* was the most highly expressed gene, *GOT1* gene was expressed in higher levels than *GOT2*, *CDO1* gene was detected in most cases, and *SLC1A1* and *SLC7A11* were expressed in similar levels. (**D**) *CDO1* mRNA expression increased in NSCLC metastatic samples compared to primary tumors. Statistical significance is represented as mean ± SD. *** *p* < 0.001, **** *p* < 0.0001.

We explored TCGA transcriptomic data considering the two most prevalent groups of NSCLC, LUAD and LUSC, and normal lung tissue to evaluate the expression dynamics of genes involved in cysteine metabolism. Regarding the genes encoding enzymes, the analysis indicated that the expression of *CBS*, *GOT1*, and *GOT2* increased in LUAD and LUSC compared to normal lung (Figure 5A,B). *CTH* was overexpressed in LUAD compared to normal lung (Figure 5A,B). No differences were found in *MPST* expression in NSCLC groups compared to normal lung tissue (Figure 5A,B). The expression of genes encoding the transporters EAAT3 (*SLC1A1*) and xCT, (*SLC7A11*) decreased and increased, respectively, in both LUAD and LUSC compared to normal lung (Figure 5A,B). *CDO1* was found downregulated in both LUAD and LUSC in comparison to normal tissue (Figure 5A,B).

The mRNA from a cohort of 55 NSCLC patients followed in IPO-Lisboa was used to quantify the expression of *CBS*, *CTH*, *MPST*, *GOT1*, *GOT2*, *CDO1*, *SLC1A1*, and *SLC7A11* genes. Correlations and associations between gene expression, tumor histotypes, and somatic mutations were explored. Regarding genes encoding H_2_S-producing enzymes (*CBS*, *CTH*, and *MPST*), *MPST* was the most expressed gene compared to *CBS* and *CTH* (Figure 5C). *GOT1* showed higher expression levels than *GOT2*, and *CDO1* was detected in most cases (Figure 5C). The genes encoding EAAT3 (*SLC1A1*) and xCT (*SLC7A11*) transporters were expressed in quite similar levels among cases (Figure 5C). Comparing the expression of genes in NSCLC primary tumors and metastases, only *CDO1* mRNA levels were significantly increased in metastatic samples (Figure 5D). Considering the correlation between the expression of cysteine-related genes, a significant Spearman correlation was observed for the expression of *CBS* with *CTH*, *MPST*, *GOT1*, *GOT2,* and *CDO1*; *CTH* with *MPST*, *GOT1*, *GOT2*, *CDO1,* and *SLC7A11*; *MPST* with *GOT1*, *GOT2*, *CDO1,* and *SLC1A1*; and *GOT1* with *GOT2* (Table 3 and Supplementary Figure S2). A very strong association was found between *CBS* and *GOT1* (*r* = 0.7198), *MPST* and *CTH* (r = 0.7013), and *CTH* and *GOT1* (r = 0.7040). In an attempt to associate particular metabolic profiles with genetic status, correlations between cysteine-related genes were explored in cases presenting mutations in *EGFR* or *KRAS* genes. In the *EGFR^Mut^* group, a significant Spearman correlation was found for the expression of *CBS* with *MPST*, *GOT1,* and *GOT2*; *MPST* with *GOT2* and *SLC1A1*; *CTH* with *MPST, GOT2,* and *SLC7A11*; and *GOT1* with *SLC1A1* and *SLC7A11* (Table 3 and Supplementary Figure S2). A very strong correlation was observed between *MPST* and *CTH* (r = 0.7868), *CTH* and *GOT2* (r = 0.7521), and *SLC7A11* and *GOT1* (r = 0.7455). In the *KRAS^Mu^*^t^ group, a significant Spearman correlation was found for the expression of *CBS* with *CTH*, *MPST,* and *GOT1*; *CTH* with *MPST*, *GOT1,* and *CDO1*; *MPST* with *GOT1* and *SLC1A1*; and *GOT1* with *CDO1* (Table 3 and Supplementary Figure S2). Very strong Spearman correlations were found between *CBS* and *CTH* (r = 0.9031), *CBS* and *GOT1* (r = 0.8901), and *CTH* and *GOT1* (r = 0.9198).

## 4. Discussion

Cancer metabolism is a rediscovered research area, and it encloses several queuesto better understand cancer biology and find disease specificities, which can be targeted in novel and more personalized therapies. Cysteine metabolic circuitries have been highlighted in recent years as being pivotal in cancer metabolic rewiring, contributing to cancer cells’ survival and chemoresistance [42,57]. In this study, new insights into the metabolic adaptation and heterogeneity of NSCLC cells are presented, accounting for a more profound knowledge of different subsets of the disease. Common cancer features can go side by side with cancer cell specificities, constituting useful targets and markers. 

The metabolic reliance on cysteine was confirmed in three NSCLC cell lines, although specificities were observed and will be further discussed. All cell lines presented similar H_2_S levels (Figure 1A). Our results indicate that while A549 and H292 may rely mostly on CBS and CSE to generate H_2_S, PC-9 appears to rely mostly on the CAT/MST axis for H_2_S generation. As H_2_S steady-state levels result from a balance of its synthesis and consumption, it remains to be established whether the sulfide oxidation pathway (not directly involved in cysteine metabolism) also operates differently between cell lines. Furthermore, the metabolic reliance of these NSCLC cell lines was disclosed by considering the two main biosynthetic and bioenergetic substrates, glucose and glutamine, together with cysteine as a main player in redox control and metabolic specificities accounting for energy and biomass production. In a recent study [13], we reinforced the relevance of glucose and lactate dynamics in NSCLC, demonstrating that different NSCLC cell lines show different metabolic profiles and glucose versus lactate reliance. Lactate is no longer considered a waste product, and it is accepted as a valuable metabolic source to supply oxidative phosphorylation (OXPHOS) and gluconeogenesis [10,14,15,16,17,18], being considered a major fuel for the TCA cycle in some NSCLC tumors [18]. Beyond the production of lactate, glycolysis is a supplier of other glucose-dependent pathways, such as the PPP, which plays a role in biosynthesis and redox control [8,57]. PC-9 is the only cell line in which glucose was detected in the extracellular media (Figure 3E), meaning that its dependence on glucose may be lower compared to A549 and H292 cell lines. This observation is in accordance with the results showing that glycolysis inhibition by BPA can be compensated by the addition of cysteine in order to generate H_2_S to maintain the ATP intracellular content (Figure 1C–F) and that PC-9 cells sustain higher H_2_S levels upon BPA exposure (Figure 1C–F). 

Glutamine is posited as the main substitute of glucose to supply OXPHOS. In this case, the most common route is glutamine-derived glutamate being converted into α-ketoglutarate and entering the TCA cycle. PC-9 was the only cell line presenting glutamine in the intracellular extracts, with a trend of having lower extracellular levels of glutamine than A549 and H292 (Figure 3A), which is consistent with the lower glucose uptake. 

As mentioned above, A549 and PC-9 cells presented distinct metabolic profiles, and H292 cells showed common metabolic patterns with A549 and PC-9 cells (Figure 3G,H,IK). When forcing the separation between cell lines considering the extracellular metabolites that contribute to differences in a PLS-DA analysis, it was verified that higher levels of amino acids and lactate and lower levels of pyruvate, acetoacetate, glutamine, and choline contributed to this separation (Figure 3I,J). The separation was more evident between A549 and PC-9 cell lines (Figure 3K,L), and this can be partially related to the genetic background of these cell lines. As we described recently, PC-9 (*EGFR^Mut^*) relies more on glucose, and A549 (*KRAS^Mut^*) are more dependent on glutamine [13]. Nevertheless, the metabolic profile of PC-9 and H292 may depend more on cysteine than A549 because cysteine was detected within A549 cells but not in the other cell lines (Figure 3A). Moreover, the higher levels of intracellular glutamate in A549 cells (Figure 3A) also reinforce the fact that these cells use less glutamate for cysteine import than H292 and PC-9 because glutamate is used in the exchange with cysteine through the xCT antiporter, which is expressed in all cell lines (Figure 2B). Hence, extracellular glutamate levels were found to be higher in PC-9 supernatants than in the other cell lines. The main source of glutamate is glutamine, which was detected intracellularly in PC-9 cells; thus, its extracellular levels tended to be lower in PC-9 and H292 cells compared to A549 (Figure 3A,D). Besides this, cysteine metabolic reliance can be assessed by the ability to synthesize GSH. PC-9 and H292 cells, but not A549, had GSH in the intracellular extracts (Figure 3A). Therefore, A549 accumulated the GSH components as it was the only cell line presenting cysteine and also the highest levels of intracellular glutamate and glycine among all the cell lines (Figure 3A). GSH regeneration also occurs through PPP [58,59], a glucose-dependent biosynthetic pathway; thus, the GSH levels in PC-9 and H292 (Figure 3A) may indicate a prevalent role of glucose in biosynthesis in these cells. However, cysteine can also be a supplier of PPP as a gluconeogenic amino acid as cysteine-derived pyruvate can be used to synthesize glucose-6-phosphate, the initial substrate of PPP [60,61,62]. 

**Figure 6:**
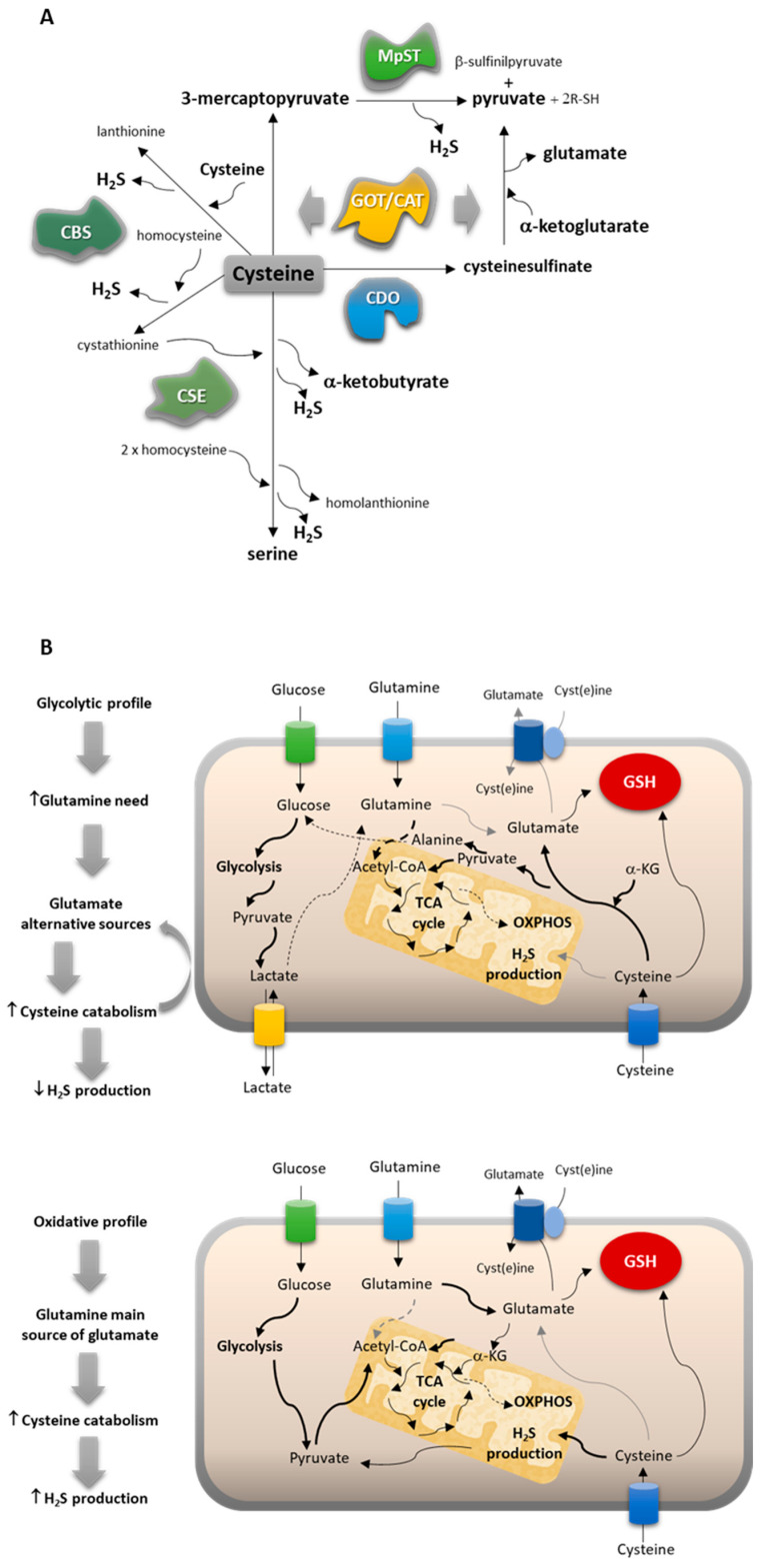
NSCLC subtypes present different metabolic profiles, adjusting their reliance on cysteine. (**A**) Cysteine catabolic pathways can be divided into pathways of cysteine enzymatic breakdown to produce H_2_S and organic intermediates mediated by cystathionine β-synthase (CBS), cystathionine γ-lyase (CSE), and/or 3-mercaptopyruvate sulfurtransferase (MST), which work together with cysteine aminotransferase (CAT/GOT), or cysteine oxidative metabolism through cysteine dioxygenase (CDO1). (**B**) Cysteine metabolism is conditioned by the glucose versus glutamine reliance of cancer cells.

By this means, cysteine can also be used to supply other metabolic pathways. In ovarian cancer, we demonstrated that pyruvate, alanine, and lactate can be cysteine derived [19]. Importantly, these compounds are gluconeogenic [8,63]. PC-9 cells presented metabolic patterns related to the gluconeogenic role of cysteine, which seemed to be more evident than in the other cell lines. Alanine can be derived from pyruvate, and alanine intracellular levels and lactate extracellular levels were significantly higher in PC-9 than in A549 and H292 (Figure 3A,E). Added to the fact that PC-9 was the only cell line that presented glucose in the extracellular media, this corroborates the synthesis of pyruvate from cysteine, at least in part. Pyruvate is afterward converted into alanine and channeled to other metabolic pathways or converted into lactate and exported outside of the cell. These observations are in accordance with the fact that PC-9 tended to be more dependent on MST enzyme than on CBS or CSE upon cysteine supplementation (Figure 1B), promoting the production of pyruvate as a product of cysteine degradation (Figure 6A). 

Furthermore, this evidence, together with the fact that PC-9 expressed higher levels of *CDO1* than the other cell lines, reinforces that cysteine may be a source of pyruvate and glutamate. CDO1 participates in the synthesis of pyruvate from cysteine, and glutamate is generated from α-ketoglutarate in this pathway [39,40] under the action of CAT/GOT enzymes (Figure 6A). This way, CDO1 may complement the action of MST as these two enzymes work in partnership with CAT enzymes, encoded by *GOT1* and *GOT2* genes, with *GOT2* demonstrated to be highly expressed in H292 and PC-9 cell lines (Figure 2C). These results are very important because CDO1 is not often considered in cancer metabolism as an intervention in cysteine-derived pyruvate production, being only explored as a mediator of taurine synthesis in NSCLC [64]. 

Concerning cysteine endogenous synthesis, the reverse transsulfuration pathway is supplied by homocysteine deviated from the methionine cycle, which, together with the folate cycle, makes up the one-carbon metabolism [65,66]. Homocysteine and homocystine were not detected in intracellular and extracellular pools of metabolites in any NSCLC cell line, and intracellular methionine levels were similar in all NSCLC cell lines (Figure 3A). However, extracellular methionine levels in PC-9 were higher compared to A549 and H292 (Figure 3D), suggesting that PC-9 depends more on the uptake than on cysteine synthesis because methionine is an essential amino acid [67] whose *de novo* synthesis cannot occur within the cells.

In a volcano plot evaluating significance versus magnitude of change, guanosine, arginine, proline, and nicotinurate came out as significant distinguishers of A549 and PC-9 cell lines (Figure 3M,N). Guanosine is a purine needed for different cellular processes, namely, the synthesis of nucleotides. In cancer, the upregulation of guanosine monophosphate synthase has been described and related to apoptosis inhibition and chemoresistance [68,69]. In SCLC, chemoresistant tumors present high levels of guanosine nucleotides [70]. However, in our study, we verified a similar pattern of chemoresistance among all cell lines (Figure 4A). Arginine and proline are precursors of glutamine and glutamate syntheses [71,72,73], and the undetectable levels of arginine and proline in PC-9 (Figure 3A) can be related to the need for glutamine and glutamate. As mentioned above in this cell line, glutamine seems to be a substitute for glucose and a precursor of glutamate, which is needed for cysteine uptake through xCT. Nicotinurate is an acylglycine, a minor metabolite of fatty acid catabolism [74]. No studies discuss the role of nicotinurate in cancer, although it has been detected in castration-resistant prostate cancer cells [75] and seems to be a stably detected compound during the carcinogenic process in gastric cancer [76]. Nicotinurate can be a product of fatty acid metabolism considering it was only detected in the A549 cell line (Figure 3N), which was also producing and exporting detectable amounts of acetoacetate (Figure 3E), a ketone body [77]. This cell line may have a different profile of fatty acid management between catabolism and anabolism, which must be addressed in the future. Little is known about the role of acetoacetate in lung cancer, but a study addressing the impact of a ketogenic diet in lung cancer xenograft showed a beneficial effect in increasing oxidative stress resistance and improving the response to chemo- and radiotherapy [78].

As a result, two main cysteine metabolic profiles can be disclosed considering the reliance on glucose versus glutamine and the way cysteine can contribute to fill metabolic gaps (Figure 6B). NSCLC cells that present a more glycolytic profile (represented by the PC-9 cell line) use glucose mainly to supply biosynthesis with an intensive production of lactate, increasing the need for glutamine to supply bioenergetics. Thus, glutamate alternative sources are needed, and cysteine can fulfill this role by following the catabolic route of CAT/MST or CDO1 and giving rise to pyruvate and glutamate. The oxidative metabolism through CDO1 does not generate H_2_S and accounts for its global decreased levels. NSCLC cells presenting a more oxidative profile (represented by the A549 cell line) use glucose to supply bioenergetics and biosynthesis. The reliance on glutamine decreases, and it works as the main source of glutamate. Cysteine catabolism occurs mainly through the H_2_S-generating pathways dependent on CBS and CSE, accounting for global increased levels of H_2_S.

Independently of the preferential cysteine catabolic pathway used by NSCLC cells, all of them are able to degrade cysteine, produce H_2_S, and maintain or increase ATP generation, even upon glycolysis inhibition (Figure 1). Therefore, given that these cell lines are chemoresistant to the main drugs used in conventional NSCLC therapy, we tested the effect of SeChry, which we had published as an inhibitor of cysteine catabolism through CBS [51]. SeChry as a new therapy could be more effective in cancer cell lines with increased antioxidant capacity, such as those presenting resistance to conventional cytotoxic drugs, as we have shown (Figure 4A), which are commonly more capable of scavenging reactive oxygen species (ROS). All the tested drugs still promote ROS generation according to published studies [79,80,81,82], even the ones that do not present the induction of ROS generation as the main mechanism of action. The platinum salts (carboplatin and cisplatin), temozolomide, and cyclophosphamide are alkylating agents, directly reacting with proteins and DNA and inducing cell injury and DNA damage as they also stimulate the generation of ROS (https://go.drugbank.com/drugs/DB00544; accessed at 21 April 2023). 

The use of a folate-conjugated dendrimer nanoparticles to deliver SeChry (SeChry@PURE_G4_-FA) was an attempt to more specifically direct nanoparticles to cancer cells as they express overall higher levels of folate receptor [56,83,84]. Interestingly, the EC_50_ for SeChry@PURE_G4_-FA was inversely proportional to the expression levels of FR-α (Figure 4B–D). Therefore, all NSCLC cell lines, except PC-9, were quite sensitive to SeChry and SeChry@PURE_G4_-FA. PC-9 presented an EC_50_ for SeChry, very close to noncancer cell lines (Figure 4B–D), in line with PC-9’s metabolic reliance on cysteine not depending on CBS. It remains to be clarified why free SeChry had slightly lower EC_50_ in NSCLC cell lines compared to encapsulated SeChry as we have previously described in ovarian cancer [51,56]. Despite this, the nanoformulation SeChry@PURE_G4_-FA can still be a good option for a SeChry-based therapy as it protects noncancer cells from SeChry toxicity, as we saw by the lack of effect of SeChry@PURE_G4_-FA on HK2 and HaCaT (Figure 4D). Moreover, free or encapsulated SeChry impaired the proliferation of NSCLC cell lines (Supplementary Figure S1), which constitutes an important feature in cancer therapy. 

Bringing cysteine metabolic circuitries to the NSCLC context may open new perspectives on disease management with the identification of new and more specific targets and markers. Therefore, the expression of pivotal players in cysteine transport and catabolism was explored in the TCGA database. In LUAD and LUSC histotypes, compared to normal lung tissue, *CBS*, *GOT1*, *GOT2,* and *SLC7A11* were overexpressed; *SLC1A1* and *CDO1* were downregulated; and *MPST* expression was not altered. Additionally, *CTH* was overexpressed in LUAD but not LUSC, thereby representing a clear distinctive pattern between the two histotypes that can be further explored. The proteins encoded by the overexpressed genes can serve as therapeutic targets, as we have demonstrated using SeChry, a well-tolerated compound by noncancer cells and an effective cytotoxic and growth controller in NSCLC depending on CBS activity, such as A549 and H292 cell lines. The proteins encoded by these genes can also be validated as therapy response markers as cysteine metabolic reliance accounts for chemoresistance, and they will function as an important tool to predict disease prognosis. Downregulated genes, such as *SLC1A1* and *CDO1*, can help to find specificities that are exhibited less frequently in NSCLC cases. This was the case for *CDO1*, whose dependence was shown by the PC-9 cell line presenting a versatile cysteine reliance pattern. The results suggest that PC-9 cells use CDO1 to oxidatively degrade cysteine in order to sustain pyruvate and glutamate levels and MST to sustain cysteine-dependent H_2_S generation and account for pyruvate yield. 

Importantly, we addressed the expression profile of cysteine-related genes in a cohort of NSCLC patients characterized for the prevalent somatic mutations found in NSCLC, affecting *EGFR* and *KRAS* genes. Some in vitro profiles were reinforced by the expression levels of cysteine-related genes in NSCLC samples. *MPST* was the most expressed cysteine-degrading and H_2_S-producing enzyme encoding a gene (Figure 5C), and strong Spearman correlations (0.4 < r < 0.69) with statistical significance (*p* < 0.01) were observed between *MPST* expression and its metabolic partners *GOT1* and *GOT2* as well as *CDO1*, whose encoded enzyme acts sequentially with *GOT1-* and *GOT2-*encoded enzymes (Table 3 and Supplementary Figure S2). This pattern of genes related to cysteine fits with the one observed in the PC-9 cell line. The relevance of MST action in cancer requires clarification [85], and our study provides one more step toward this. The significant correlations of *MPST* with *GOT2*, *SLC1A1,* and *SLC7A11* and *MPST* with *GOT1* and *SLC1A1* were also verified in *EGFR^Mut^* and *KRAS^Mut^* tumors, respectively (Table 3 and Supplementary Figure S2), suggesting that cysteine catabolism routes through MST and CAT/GOT enzymes with the production of H_2_S and through CDO1 and CAT/GOT enzymes are pivotal in some subsets of NSCLC. Moreover, *CDO1* was significantly more expressed in NSCLC metastases than in primary tumors (Figure 5D). Contrary to what has been shown by different studies indicating *CDO1* may be a tumor suppressor gene [86,87,88,89,90,91], our results point out that its function may contribute to more aggressive phenotypes. This indicates that there is an opportunity for therapeutic intervention by targeting CDO1 in particular cancer contexts in which *CDO1* silencing is not occurring as it was described that *CDO1* silencing by methylation was occurring preferentially in *KEAP1-*mutated cases in NSCLC [64]. KEAP1 is a negative regulator of NRF2, the main controller of oxidative stress. Therefore, upon KEAP1 inactivation, the overexpression of genes regulated by NFR2 will occur, including *SLC7A11* [92]. No significant correlations were observed between the expression of *CDO1* and *SLC7A11* genes (Table 3 and Supplementary Figure S2). Hence, more studies are needed to ensure CDO1 targeting can be a suitable strategy to treat some subsets of NSCLC. 

Considering the EAAT3 and xCT transporters, their encoding genes (*SLC1A1* and *SLC7A11,* respectively) were expressed at similar levels in NSCLC samples (Figure 5C), suggesting that cysteine enters the cell as a free amino acid and also as a dimer, cystine, because EAAT3 is a cysteine transporter and xCT is a glutamate/cystine antiporter [57]. Interestingly, the expression of *SLC1A1* presented a significantly strong Spearman correlation with the expression of *MPST* and *GOT1* in the *EGFR^Mut^* group, while in all NSCLC cases and in *KRAS^Mut^* samples, the correlation was significant only with *MPST* expression (Table 3 and Supplementary Figure S2). The expression of *SLC7A11* in all NSCLC cases and in the *EGFR^Mut^* group presented a significantly strong correlation with *GOT1* expression, while in the *EGFR^Mut^* group, a significantly strong correlation was also observed with *MPST* expression (Table 3 and Supplementary Figure S2). A significant moderate correlation between *SLC7A11* and *CTH* expression was observed in all NSCLC cases (Table 3 and Supplementary Figure S2). The relevance of xCT function in chemoresistance and cell death inhibition, namely, through ferroptosis, is already set in NSCLC [92,93], and our study is in accordance with this, reinforcing that xCT blockade can be a useful adjuvant therapy to improve the efficacy of conventional cancer therapy.

Besides our results highlighting the role of MST and CDO1 in cysteine metabolism, the correlations observed between *CBS* and *CTH* with other genes encoding enzymes and transporters support the role of CBS and CSE on cancer biology disclosed in other studies [45,46,94,95,96], as we also verified in our H_2_S detection assays using A549 and H292 cell lines (Figure 1). Interestingly, in *KRAS^Mut^* and WT tumor samples, a correlation was observed between *CBS* and *CTH* genes expression, corresponding to A549 and H292 genetic background (Table 3 and Supplementary Figure S2), while the expression of *CBS* and *CTH* were not correlated in PC-9 cells (*EGFR^Mut^*). Again, the genetic background of tumors must be explored and comprehensively correlated with metabolic profiles with the aim of finding mechanisms of resistance to target therapy applied in NSCLC.

The definition of cancer metabolome will hardly allow clear-cut identification of markers for disease. However, the definition of profiles that fit certain clinical behaviors and patient subsets can constitute a powerful tool in the prognosis and stratification of patients who may benefit from new therapies. Heterogeneity defines individual profiles, and the identification of new targets among metabolic players is also a step forward in cancer management toward personalized medicine.

## Figures and Tables

**Table 1:** Genes analyzed; the respective primers used in RT-PCR; and the corresponding features, namely, primer length, annealing temperature (AT), GC percentage (%), and fragment length.

Gene	Primer	Sequence (5′→3′)	Length (nt)	AT (°C)	GC%	Fragment (bp)
*Cystathionine beta-synthase (CBS; GC21M043053)*	Forward	GAGCTCTTGGCCAAGTGTG	19	60	58	232
	Reverse	GCACGTCCACCTTCTCGG	18		67	
*Cystathionine Gamma-Lyase (CTH; GC01P070411)*	Forward	GCAGCCACTGTAACTATTACCC	22	60	50	175
	Reverse	CTGGTGTAATTGCTGCCTCTAG	22		50	
*Mercaptopyruvate Sulfurtransferase (MPST, GC22P037019)*	Forward	CTTCATCAAGACCTACGAGGAC	22	60	50	134
	Reverse	GGTAGTGGCCAGGTTCAATG	20		55	
*Glutamic-Oxaloacetic Transaminase 1 (GOT-1; GC10M099396)*	Forward	GAGAAGAGAGGATTGGACCTC	21	60	52	147
	Reverse	CATGACAGAAGCAATCTGCTTCC	23		48	
*Glutamic-Oxaloacetic Transaminase 2 (GOT-2, GC16M058707)*	Forward	CCAGAGCCAGCTCCTGGT	18	61	67	171
	Reverse	CTGCCTTGCGGACGCTAG	18		67	
*Solute Carrier Family 7 Member 11 (SLC7A11; GC04M138164)*	Forward	GGTCCTGTCACTATTTGGAGC	21	61	58	136
	Reverse	GAGGAGTTCCACCCAGACTC	20		59	
*Solute Carrier Family 1 Member 1 (SLC1A1; GC09P004490)*	Forward	GTATCACGGCCACATCTGCC	20	61	60	121
	Reverse	GCAATGATCAGGGTGACATCC	21		52	
*Hypoxanthine-guanine phosphoribosyltransferase (HPRT1; GC0XP134460)*	Forward	TGACACTGGCAAAACAATGCA	21	58	43	94
	Reverse	GGTCCTTTTCACCAGCAAGCT	21		52	
*Cysteine dioxygenase 1 (CDO1; GC05M115804)*	Forward	GAGACATTATTTGCCTGGC	19	56	47	149
	Reverse	CTCACAGCAGGTTCCGTAT	19		53	

**Table 2:** Proteins detected by immunofluorescence and the antibodies used.

Target Protein	Antibody Reference
*CBS*	WH0000875M1, Sigma-Aldrich
*CSE*	SAB1403711, Sigma-Aldrich
*MST*	HPA001240, Sigma-Aldrich
*xCT*	ab175186, Abcam; Cambridge, UK
*EAAT3*	14501S, Cell Signaling Technology; Danvers, MA, USA

**Table 3:** Spearman correlation between the expression of metabolic genes in NSCLC samples considering the total cases and cases presenting *EGFR* and *KRAS* mutations, *EGFR^Mut^* and *KRAS^Mut^*. Significant correlation was considered at *p* < 0.05; ns—not significant.

NSCLC total cases									
**Gene**		*CBS*	*CTH*	*MPST*	*GOT1*	*GOT2*	*SLC1A1*	*SLC7A11*	*CDO1*
		*CBS*	*r* = 0.6885 *p* = <0.0001	*r* = 0.6405 *p* = <0.0001	*r* = 0.7198 *p* = <0.0001	*r* = 0.3672 *p* = 0.0068	ns	ns	*r* = 0.4127 *p* = 0.0021
			*CTH*	*r* = 0.7013 *p* = <0.0001	*r* = 0.7040 *p* = <0.0001	*r* = 0.5951 *p* = <0.0001	ns	*r* = 0.3608 *p* = 0.0080	*r* = 0.4596 *p* = 0.0005
				*MPST*	*r* = 0.6183 *p* = <0.0001	*r* = 0.4593 *p* = 0.0005	*r* = 0.6254 *p* = <0.0001	ns	*r* = 0.4255 *p* = 0.0015
					*GOT1*	*r* = 0.4537 *p* = 0.0006	ns	*r* = 0.4574 *p* = 0.0006	ns
						*GOT2*	ns	ns	ns
							*SLC1A1*	ns	ns
								*SLC7A11*	ns
									*CDO1*
NSCLC *EGFR^Mut^* and *KRAS^Mut^* cases									
**Gene**		*EGFR* ^Mut^							
		*CBS*	*CTH*	*MPST*	*GOT1*	*GOT2*	*SLC1A1*	*SLC7A11*	*CDO1*
*KRAS* ^Mut^	*CBS*		ns	ns	*r* = 0.5947 *p* = 0.0352	ns	ns	ns	ns
	*CTH*	*r* = 0.9031 *p* = <0.0001		*r* = 0.7868 *p* = 0.0021	ns	*r* = 0.7521 *p* = 0.0041	ns	ns	ns
	*MPST*	*r* = 0.6481 *p* = 0.0027	*r* = 0.6466 *p* = 0.0028		ns	*r* = 0.6437 *p* = 0.0202	*r* = 0.6429 *p* = 0.0208	*r* = 0.5604 *p* = 0.0499	ns
	*GOT1*	*r* = 0.8901 *p* = <0.0001	*r* = 0.9198 *p* = <0.0001	*r* = 0.6980 *p* = 0.0009		ns	*r* = 0.6355 *p* = 0.0224	*r* = 0.7455 *p* = 0.0047	ns
	*GOT2*	ns	ns	ns	ns		ns	ns	ns
	*SLC1A1*	ns	ns	*r* = 0.6326 *p* = 0.0048	ns	ns		ns	ns
	*SLC7A11*	ns	ns	ns	ns	ns	ns		ns
	*CDO1*	ns	*r* = 0.5564 *p* = 0.0165	ns	*r* = 0.5308 *p* = 0.0234	ns	ns	ns	

## Data Availability

The datasets generated and analyzed during the current study are available in the GitHub repository, https://github.com/lgafeira/H2S_cancer, accessed at 16 July 2023.

## Supplementary Materials

The following supporting information can be downloaded at: https://www.mdpi.com/article/10.3390/antiox13010051/s1, Figure S1: SeChry and SeChry@PUREG4-FA induced significant decrease in cell proliferation across the NSCLC panel used; Figure S2: (A) Spearman correlation was significant for the expression of several analyzed genes: *CBS* with *CTH*, *MPST*, *GOT1*, *GOT2,* and *CDO1*; *CTH* with *CBS*, *MPST*, *GOT1*, *GOT2*, *CDO1,* and *SLC7A11*; *MPST* with *CBS*, *CTH*, *GOT1*, *GOT2*, *CDO1,* and *SLC1A1*; and *GOT1* with *GOT2* and *SLC7A11.* (B) Correlations between cysteine-related genes in cases presenting *EGFRMut* indicated a significant Spearman correlation for the expression of *CBS* with *GOT1*; *CTH* with *MPST* and *GOT2*; *MPST* with *GOT2*, *SLC1A1,* and *SLC7A11*; and *GOT1* with *SLC1A1* and *SLC7A11.* (C) Correlations between cysteine-related genes in cases presenting *KRASMut* indicated a significant Spearman correlation for the expression of *CBS* with *CTH*, *MPST,* and *GOT1*; *CTH* with *CBS*, *MPST*, *GOT1,* and *CDO1*; *MPST* with *CBS*, *CTH*, *GOT1,* and *SLC1A1*; and *GOT1* with *CDO1.*
