# Supplementary material for: H2S-Synthesizing Enzymes Are Putative Determinants in Lung Cancer Management toward Personalized Medicine
Source: Antioxidants (Basel). 2023 Dec 28;13(1):51. doi: 10.3390/antiox13010051 (PMC10812562; doi:10.3390/antiox13010051)
Supplement: Supplementary file 1 [file antioxidants-13-00051-s001.zip › antioxidants-2783812-supplementary.pdf]

Supplementary Figures

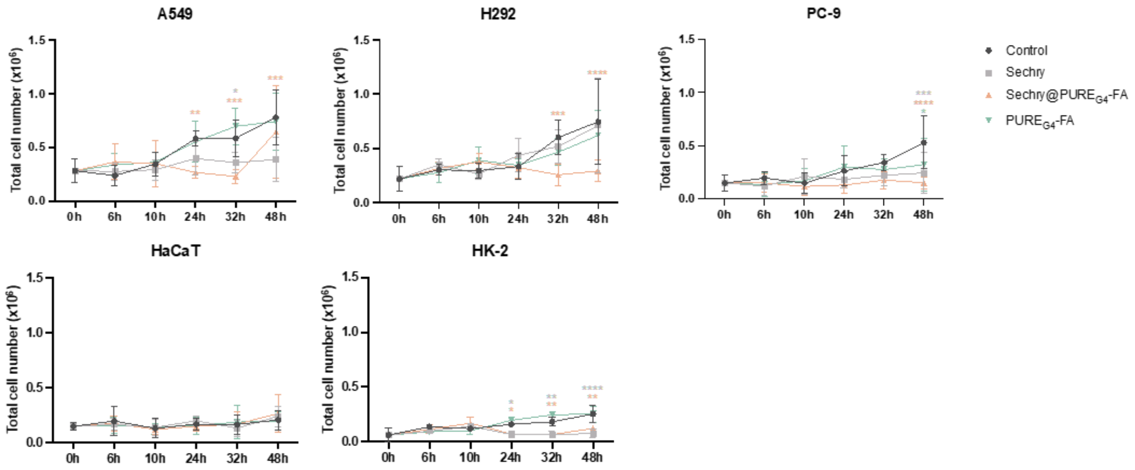

Suppl Figure 1. SeChry and SeChry@PURE$_{G4}$-FA induced significant decrease in cell proliferation across the NSCLC panel used. All data are normalized to the control condition and represented as mean ± SD. *p<0.5, **p<0.01, ***p<0.001, ****p<0.0001.

A

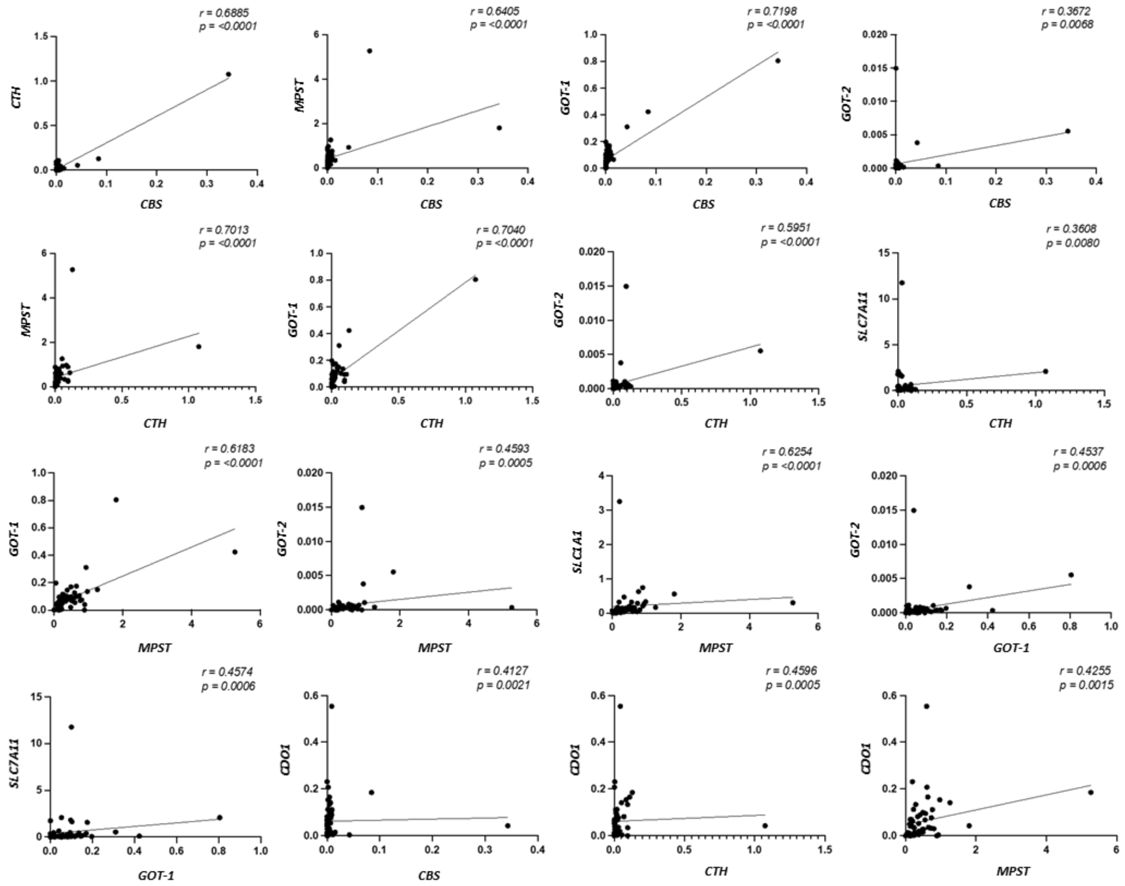
r = 0.6885
p = <0.0001
CTH
CBS
r = 0.6405
p = <0.0001
MPST
CBS
r = 0.7198
p = <0.0001
GOT-1
CBS
r = 0.3672
p = 0.0068
GOT-2
CBS
r = 0.7013
p = <0.0001
MPST
CTH
r = 0.7040
p = <0.0001
GOT-1
CTH
r = 0.5951
p = <0.0001
GOT-2
CTH
r = 0.3608
p = 0.0080
SLC7A11
CTH
r = 0.6183
p = <0.0001
GOT-1
MPST
r = 0.4593
p = 0.0005
GOT-2
MPST
r = 0.6254
p = <0.0001
SLC1A1
MPST
r = 0.4537
p = 0.0006
GOT-2
GOT-1
r = 0.4574
p = 0.0006
SLC7A11
GOT-1
r = 0.4127
p = 0.0021
CDO1
CBS
r = 0.4596
p = 0.0005
CDO1
CTH
r = 0.4255
p = 0.0015
CDO1
MPST

B

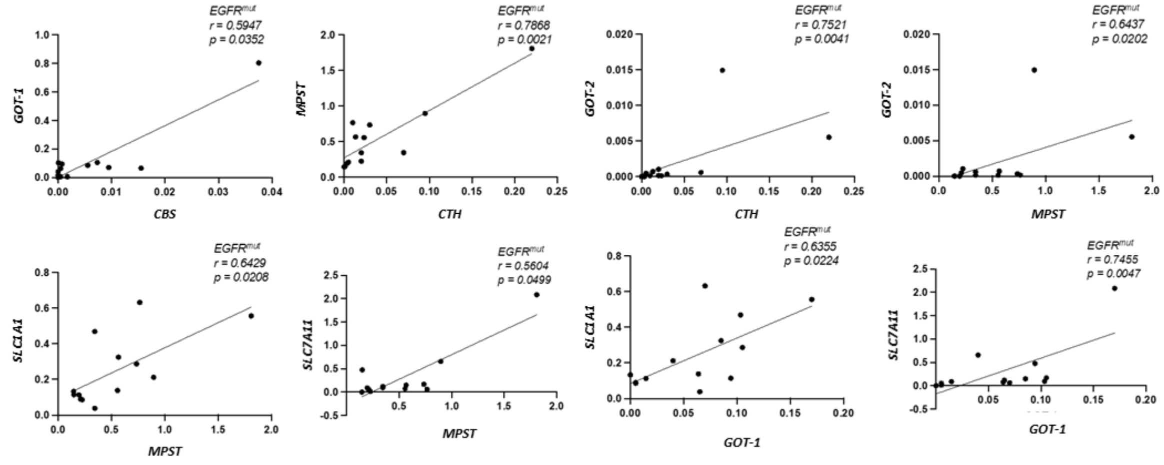
EGFR^mut
r = 0.5947
p = 0.0352
GOT-1
CBS
EGFR^mut
r = 0.7868
p = 0.0021
MPST
CTH
EGFR^mut
r = 0.7521
p = 0.0041
GOT-2
CTH
EGFR^mut
r = 0.6437
p = 0.0202
GOT-2
MPST
EGFR^mut
r = 0.6429
p = 0.0208
SLC1A1
MPST
EGFR^mut
r = 0.5604
p = 0.0499
SLC7A11
MPST
EGFR^mut
r = 0.6355
p = 0.0224
SLC1A1
GOT-1
EGFR^mut
r = 0.7455
p = 0.0047
SLC7A11
GOT-1

C

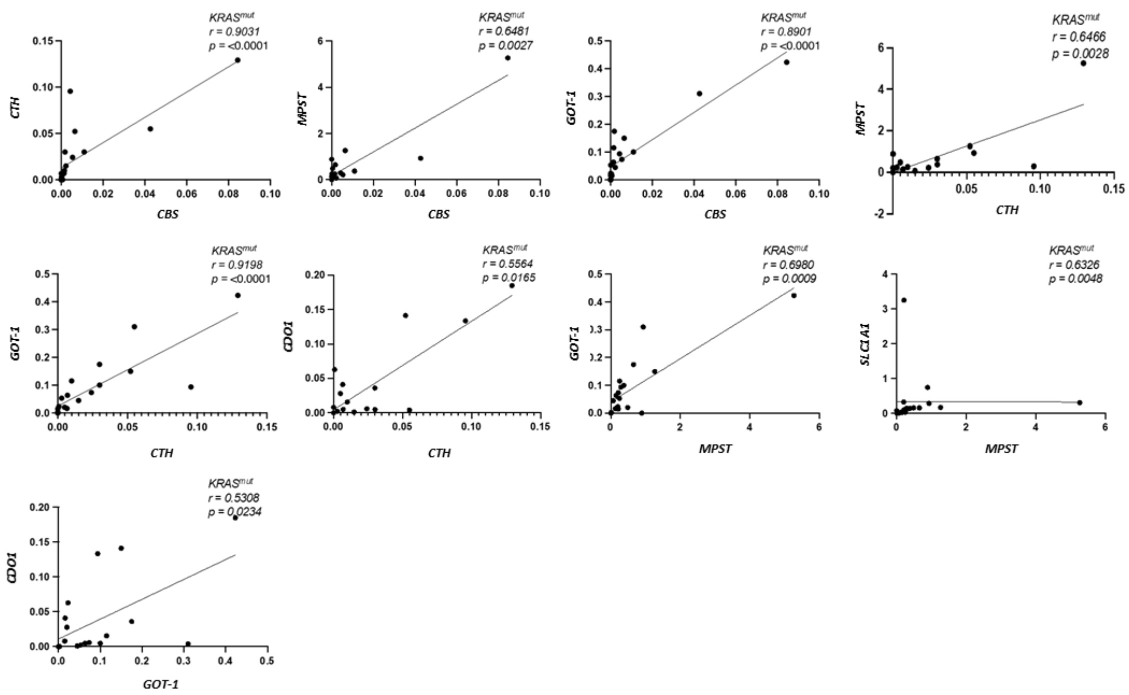

Suppl Figure 2. (A) Spearman correlation was significantly found between the expression of several analyzed genes: *CBS* with *CTH, MPST, GOT1, GOT2* and *CDO1; CTH* with *CBS, MPST, GOT1, GOT2, CDO1* and *SLC7A11; MPST* with *CBS*, *CTH*, *GOT1, GOT2, CDO1* and *SLC1A1,* and *GOT1* with *GOT2* and *SLC7A11.* (B) Correlations between cysteine-related genes in cases presenting $EGFR^{Mut}$ indicate a significant Spearman correlation between the expression of *CBS* with *GOT1; CTH* with *MPST* and *GOT2; MPST* with *GOT2, SLC1A1* and *SLC7A11,* and *GOT1* with *SLC1A1* and *SLC7A11.* (C) Correlations between cysteine-related genes in cases presenting $KRAS^{Mut}$ indicate a significant Spearman correlation between the expression *CBS* with *CTH, MPST* and *GOT1; CTH* with *CBS*, *MPST, GOT1* and *CDO1; MPST* with *CBS*, *CTH*, *GOT1* and *SLC1A1,* and *GOT1* with *CDO1.*
